# Cooperation as the Secret Ingredient in the Recipe to Foster Internal Technological Eco-Innovation in the Agri-Food Industry

**DOI:** 10.3390/ijerph17072588

**Published:** 2020-04-10

**Authors:** Adrián Rabadán, Ángela Triguero, Ángela Gonzalez-Moreno

**Affiliations:** 1 Higher Technical School of Agricultural and Forestry Engineering, University of Castilla-La Mancha, 02071 Albacete, Spain; 2 Faculty of Economics and Business Administration, University of Castilla-La Mancha, 02071 Albacete, Spain; Angela.Triguero@uclm.es (Á.T.); angela.gonzalez@uclm.es (Á.G.-M.)

**Keywords:** eco-innovation, eco-product innovation, eco-process innovation, QCA, agri-food

## Abstract

Although eco-innovation in the agri-food sector is receiving increasing amounts of attention, there is a lack of information about the specific conditions that encourage firms to develop eco-innovation strategies internally. Our empirical method relies on the data of Spanish firms operating in the agri-food sector, and uses the Qualitative Comparative Analysis (QCA). Specifically, we identify the recipes of antecedent conditions that effectively foster the internal development of technological eco-innovation, and then we analyze whether differences exist in the internal development of product and process eco-innovations. The results show that different combinations of conditions can yield internally developed eco-innovation, but all of them indicate that cooperation with stakeholders is the key to fostering technological eco-innovation in this industry. This conclusion encourages the creation of policies and incentives to promote cooperation in order to improve the sustainability of the sector.

## 1. Introduction

Firms’ environmental responsibility is in today’s conversations among practitioners and on policy makers’ agendas. Managers and entrepreneurs are everyday becoming more aware of the consequences of their firms’ activities on the environment. This environmental consciousness is also fostered from external pressures from stakeholders, such as regulators or consumers [1]. Additionally, some firms recognize that environmentally friendly behavior can lead them to obtain and maintain competitive advantages [2]. Therefore, some firms try to become greener by introducing changes in their products and manufacturing processes, enabling more efficient and responsible use of natural resources and energy. This behavior leads to the development and adoption of the so-called technological eco-innovations that take the form of new environmentally friendly products or processes.

The academic literature on the drivers of eco-innovation has been increasing lately [2,3,4], and three main drivers have been identified: the market pull [5], the regulatory push/pull [6], and the technology push [7]. However, several gaps have been found. First of all, the literature tends to avoid small- and medium-sized enterprises (SMEs) with very few exceptions [1,8,9]. Research has mainly focused on high-tech industries and on large corporations [10]. Usually, SMEs find it difficult to convert environmentally friendly practices into competitive advantages [11] and, hence, are reluctant to include environmental concerns in their management practices [12]. Secondly, the agri-food sector, more than any other industry, is characterized by a significant dependence on natural resources [13]. However, although some agri-food companies are currently introducing eco-innovation strategies into their business models [14], there is a lack of research on this topic in traditional sectors, such as the agri-food industry that are typically characterized as low-tech with notable exceptions [1,15,16,17].

A distinctive feature in the firms operating in the agri-food industry is the fact that although technological innovation has been found to be critical in these companies, especially for co-operatives [18], the sector has low research and development (R&D) intensity, while producing a significant number of innovations [19]. Additionally, most innovations in this industry are more incremental than radical—that is, these innovations are improvements to new food products, or variations of existing ones [20]. Acosta, et al. [21] argued that firms in this particular industry are aware of external sources of knowledge, including business relationships, a well-developed inter-industry network, and equipment and material purchases. As a result, food firms take advantage of external knowledge and might have no need to generate this knowledge through internal R&D expenditures. Hence, most of the innovations and eco-innovations that are adopted by firms in the agri-food industry are acquired from external sources [1], and a few are internally developed.

Therefore, there is a lack in the literature of papers that focus on SMEs and on low-tech industries, such as the agri-food industry. Eco-innovation, aimed at providing new business models, products, and manufacturing processes which incorporate new environmentally friendly formats and materials, such as bottling and packaging technology, is considered one of the research priorities for agri-food research, at least in Spain [22]. However, recent literature reviews on eco-innovation have found no single paper that explains internal development of eco-innovation in SMEs in the agri-food industry [2,23,24]. The present paper seeks to fill this gap in the literature, and this is its main contribution.

Additionally, it is important to differentiate between eco-product and eco-process innovations in this industry. In this respect, the former increases the demand for new food products, while the latter reduces the use of energy, materials, and/or natural resources in the manufacturing processes, thereby increasing the firm’s productivity and competitiveness [16]. Thus, material recycling, energy recovery, waste management, solid waste collection, and water pollution abatement are considered as examples of process eco-innovations, while organic production and the development of more sustainable food production systems would be treated as eco-product innovations.

Hence, the purpose of this paper is to determine which factors lead to the internal development of eco-innovation. By means of Qualitative Comparative Analysis (QCA), the present paper will explore the different factors that determine the effects of firms’ resources, capabilities, and cooperative activities on the internal development of eco-innovations in the agri-food industry in Spain. This particular method is suitable for research with small data samples, yet it allows for the generalization of the results, conclusions, and implications.

Consequently, in this paper we attempt to answer the following research questions:

(1) What drives the internal development of eco-innovations by agri-food SMEs considered the limited access to knowledge and resource in a mature and low-tech industry? In other words, is it possible to develop eco-innovations internally in a mature and traditional industry made up of small companies without financial resources or internal R&D?
(2) How do financial resources and profit levels influence internal adoption of eco-innovation? What is the role of technological and organizational capabilities of the firm? What is the influence of knowledge cooperation to foster internal development of eco-innovations?
(3) What are the “optimal” combinations of resources and capabilities to boost the adoption of internal development of eco-innovations by these SMEs?
(4) How will the factors interact with each other and how will the interactions affect the overall performance of eco-product and process innovations by agri-food SMEs?
(5) Does the adoption of eco-product and process innovations require similar or different combinations? Is there any interdependence between both types of eco-innovation? What is the key factor to enhance each type of eco-innovation in the food industry?

This paper contributes to the literature by focusing on internal development of eco-innovation in a low-tech industry, by differentiating between eco-product and eco-process innovations, and by applying QCA, which permits research with small data samples.

The paper is structured as follows. In Section 2, we present the literature review and the theoretical background, which are followed by the methodology in Section 3. Then, we show the results of our empirical analysis (Section 4) and we finish in Section 5 with the conclusions, implications, and limitations of our study.

## 2. Theoretical Framework

Eco-innovation is defined in the Oslo Manual as “the production, assimilation, or exploitation of a product, production process, service, or management or business method that is novel to the organization (developing or adopting it) and which results, throughout its life cycle, in a reduction of environmental risk, pollution, and other negative impacts of resource use (including energy use) compared to relevant alternatives” [25] (p. 8). Attending to this definition, we can distinguish between technological and non-technological eco-innovation. The former refers to eco-product and eco-production processes, while the latter refers to management, marketing, or business methods that reduce the impact of the firm’s activities on the environment.

The literature on eco-innovation has increased exponentially [24]. While some of the recent papers deal with the relationship between eco-innovation and other emerging topics, such as circular economy [26,27], the majority of the highly cited papers on the topic (see Table 1) are focused on the identification of drivers and factors that foster the development and adoption of eco-innovation.

**Figure 1:**
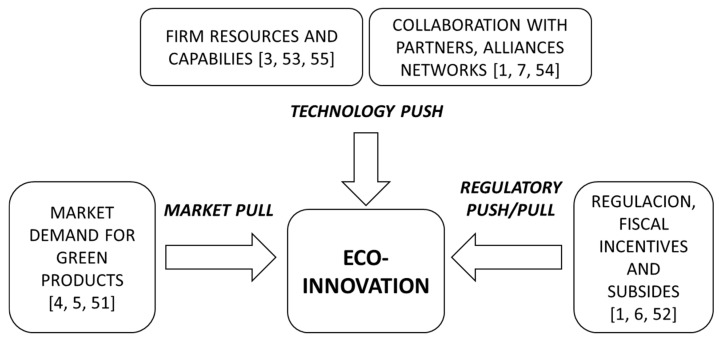
Drivers of eco-innovation. Adapted from Triguero et al. [16].

The literature on the drivers of eco-innovation has been increasing [2,3,4] and three main drivers have been identified: the market pull [4,5,51], the regulatory push/pull [1,6,52], and the technology push [1,3,7,53,54,55] (see Figure 1).

Recent research has shown a clear openness of consumers toward product innovation in the agri-food industry [56]. An increase in the consumer demand for greener products and services and an increased willingness to pay extra for environmentally friendly products and/or services has been identified as a *market pull* towards eco-innovation [16,51].

On the other hand, the literature has argued that the use of fiscal incentives and subsidies fosters the introduction of eco-innovation, thus making its benefits higher than the costs of paying fines to governments for non-compliance [52]. Additionally, *regulation* has enabled the agri-food industry to address a prominent issue involving the processing of waste materials, as well as sustainable production systems [13].

Finally, the *technology push* is also considered another key driver of eco-innovation in this industry. A firm’s resources and capabilities enable them to develop the necessary knowledge base to promote eco-innovations [53]. The role of the technology push could also come from the creation of technological networks through which firms collaborate with stakeholders, such as clients, suppliers, and universities [7,54]. This is especially relevant for SMEs.

There has been a recent call for studying SMEs’ openness and their knowledge networks [24], as they constitute a key element fostering eco-innovation that deserves further analysis [16]. In this line, some of the latest research has been focusing on the relationship between the cooperation strategies of firms and the adoption and development of eco-innovations [36,57,58]. Regarding the agri-food industry, to the best of our knowledge, only a few studies have investigated the specific drivers of the eco-innovations in this particular sector. Recently, Triguero, Fernández and Sáez-Martinez [16] proposed the framework in Figure 1 to study the influence of open innovation strategies on the adoption of radical and incremental eco-innovations in this industry. This approach is somehow similar to the framework proposed by Marotta and Nazzaro [59] on the issue of the determinants of value creation processes on farms, and further developed by Marotta and Nazzaro [13] on their recent analysis of value creation in wineries. Triguero et al. [16] concluded that customer pressure fosters eco-innovation and high standards and requirements related to food safety. Regarding firms’ R&D resources, their findings are not conclusive. Similarly, Cuerva, Triguero-Cano and Córcoles [1] corroborated part of the proposed framework by comparing eco-friendly and non-eco-friendly innovations in the Spanish food and beverage industry. They showed that these three driving factors exercised different influences on eco-product and eco-process innovations compared to non-environmental agri-food firms. Additionally, Bossle, De Barcellos and Vieira [15], in their analysis of the Brazilian food industry, proposed a relatively different framework distinguishing between internal (e.g., resources) and external factors (e.g., collaboration with partners). Finally, although not focused on eco-innovation, Cainelli, Mazzanti, and Zoboli [60] stresses the influence of cooperation in the French food industry for the development of innovations.

Based on the proposed framework in Figure 1 and considering that the market and regulatory factors are equal and constant for all firms in this industry, we will focus on the technology push factors. Therefore, we can argue that the firm’s resources, capabilities, and cooperation with stakeholders will make a difference in fostering eco-innovation in this particular sector. Hence, we will analyze the diverse combinations of resources, capabilities, and cooperation activities that result in the development of eco-product and eco-process innovations in the agri-food industry.

Despite recent efforts on the role of internal factors on eco-innovation, such as environmental management [52], skilled personnel [57], equipment renewal [5], technological capabilities [4], or cooperation [61], research on their influence is still very limited [62].

According to the resource-based view (RBV) of the firm [63,64] and from a Dynamic Capabilities perspective [65], certain firm resources and capabilities (valuable, rare, and imperfectly imitable) may be required to successfully develop and adopt eco-innovations. Therefore, the RBV provides an appropriate theoretical basis for analyzing eco-innovation, although the literature shows that there is an overlap between eco-innovation and general innovation processes [66]. A firm’s eco-innovation capacity will be connected to the pool of knowledge, resources, and capabilities that is available within the company [67]. However, most research on the topic addresses firms’ resources and capabilities that are not specific to eco-innovation and often not internally differentiated [68]. In this paper, we will explain why some firms internally develop eco-innovation through the analysis of the combinations of resources and capabilities that increase their eco-innovation performance.

In this sense, Horbach [3] contends that internal R&D, high investment intensity, and improvements in a company´s innovative capacity are important drivers of eco-innovation, since the “*availability of greater technical knowledge within a company moderates its vulnerability in the face of the demands of new environmental regulations*” [69] (p. 307). Eco-innovative activity depends directly on R&D activity, which is influenced by past activities (dependence on the technological trajectory) and activities of other companies in the same industry/sector. The empirical literature is not conclusive. While some empirical works show that R&D is essential for all types of eco-innovation [70], other research focused on the food industry finds inconclusive results [16]. Although R&D investment is considered to be a source for eco-innovation [71] that provides firms with a competitive advantage in it [72], the influence of technological capabilities on eco-innovation processes and its causal relationship has not been thoroughly elucidated to date [2].

Furthermore, apart from technological capabilities, eco-innovation activities will require the firm to have access to financial resources [53], just as any other type of innovation does. The lack of financial resources is one of the barriers to eco-innovation that is identified in the literature [72]. Having access to one’s own financial resources or to private or public funding will allow the firm to conduct the necessary investments to internally develop environmental innovations as the availability of financial resources themselves or financial slack influences eco-innovation [32]. In this sense, own-financing will allow firms to approach their eco-innovation activities with greater independence [73].

Regarding profitability, Przychodzen and Przychodzen [74] studied the relationship between the financial performance and eco-innovation activities of a sample of Polish and Hungarian firms. According to them, eco-innovative companies have lower profiles of exposure to financial risk. “*The information asymmetries could imply that the cost of financial resources increases and spreads due to a worsening in profitability from the higher risk level of the investments in eco-innovation”* [73] (p. 260). Hence, we can expect that higher financial performance will increase eco-innovation behavior through indebtedness and reduced financial risk.

Organizational capability is also a valuable resource to be considered as a driver of eco-innovations [5], specially, for internal development. In this sense, Environmental Management Systems and other eco-organizational innovations and their implementation create organizational capacities and lead to the development of technological eco-innovations [1].

Additionally, several studies have identified the positive effects of incorporating external knowledge, and, compared with other innovations, “*eco-innovation activities seem to require more external sources of knowledge and information*” [3] (p.523). Cooperation is of high importance for eco-innovation because of its characteristics, such as double externality, including positive spillovers. Moreover, the transition towards more sustainable production and consumption patterns necessarily involves several private and public actors in a system [75]. Eco-innovations require more cooperation than other innovations, given their systemic and complex character, and that eco-innovators have to leverage on the competences of external partners to a higher extent than other innovators [7].

Companies cooperate in order to reduce and share the risk, costs, and uncertainty that are associated with R&D activities [76,77]. External knowledge from customers, suppliers, and other agents are keys to environmental innovation [61,75]. Despite the limited number of studies on the influence of open innovation modes on eco-innovation in food firms, some interesting research shows that the use of a variety of external knowledge sources has a positive influence on eco-innovation in the manufacturing sector [70,72].

According to Acosta, Coronado, Ferrándiz, León and Moreno [21], food firms take advantage of external knowledge and might have no need to generate this knowledge through internal R&D expenditures. A distinctive characteristic of the food industry in Europe is that firms have low R&D intensity while producing a significant number of innovations [19]. The agri-food industry is dominated by SMEs, which lack knowledge on how to commercialize their own technology [78], and most innovations are mere improvements to new food products or variations of existing ones [20]. However, these firms are continuously exposed to external sources of knowledge, professional relationships, and a well-developed inter-industry network [21].

**Figure 2:**
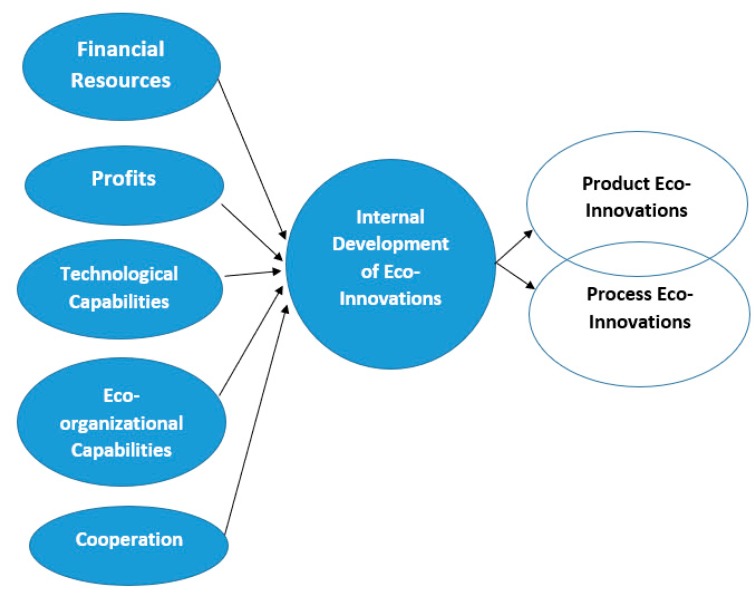
The influence of cooperative activities and the combination of resources and capabilities on the internal development of eco-innovations by firms.

This paper analyses the influence of cooperative activities and the combination of resources and capabilities on the internal development of eco-innovations by firms in a traditional industry—the agri-food industry in Spain (see Figure 2).

In our paper, we will also include two additional variables: the size and group of firms. Both reflect the firm’s availability to financial, human, and even organizational resources and capabilities due to its bigger size and/or from being part of a bigger corporation [79]. In this regard, size has also been analyzed as a source for eco-innovation [17], since larger companies are supposed to have higher levels of external financing for eco-innovation [79]. Additionally, the availability of financial resources is related to R&D, since firms will invest if they can access sufficient financing at a reasonable cost, and this availability depends, among other things, on the characteristics of the firm, such as its size [80].

An additional goal of this study is the distinction between process and product eco-innovations. Although it is true that if a company decides to put an eco-innovative product on the market, it will necessarily have to implement eco-innovative production processes, and vice versa (i.e., if the company adopts eco-innovative production processes, the final product of these eco-innovative processes will obviously be eco-innovative), there is a gap between theory and firm innovation behavior. Regarding eco-innovation performance, firms can adopt significant changes in their production processes by adopting cleaner technologies. In these cases, the final product may be more eco-friendly due to the reduction of environmental harm, but it does not mean that these firms are introducing eco-product innovations. Food firms introduce cleaner processes to reduce energy use or waste so as to increase their production efficiency through cost reduction, and they also implement End-of-Pipe technologies to comply with environmental legislation [4,11]. Both are process eco-innovations, but there are innovations related to the improvements to existing products or the development of new eco-products that achieve other purposes [81,82]. Although previous empirical research states that “*firms adopt both types of eco-innovations to improve their competitive advantage, because one type of innovation often requires the other*” [58] (p.16), the adoption of eco-product and eco-process innovation relies on different resources, capabilities, and knowledge bases. Thus, the study of the specific conditions that encourage firms to develop each type of eco-innovation is considered separately. In addition, the conditions and core competencies that foster internal development of eco-product and eco-process innovations by Spanish SME food firms are heterogeneous, due to the complexity being higher for eco-innovation than for traditional innovation [57,70]. In fact, the introduction of sustainable processes (green manufacturing) and eco-products is a major innovative trend in the food industry, but each company shows innovative performances. They neither have the resources and capabilities, nor are able to combine the resources and skills to meet the challenges involved in each type of eco-innovation in the same way.

## 3. Materials and Methods

### 3.1. Database

The food industry is one of the most important branches of the national economy in Spain and the European Union, with high relevance for employment and economic output. Spanish food firms are mainly process innovation-oriented [16]. In this industry, new technologies are developed by upstream industries, and innovation occurs through equipment and capital good investments [83].

The original sample contained the data of 277 agri-food companies operating in Spain in 2016. Taking into account the fact that the food industry is a low-tech and mature sector, the adoption of eco-processes is more habitual than the introduction of eco-products. This evidence is shown by our data. From the initial sample, 79 companies had developed technological eco-innovations (products or processes) in the last 3 years. Specifically, 21 companies had developed product eco-innovations, and 66 had developed process eco-innovations. Within the companies that developed product eco-innovations, 16 relied on internal innovations, and five acquired that innovation. For the process eco-innovations, up to 32 companies developed the processes internally, while 34 acquired the eco-innovations.

### 3.2. Methodology

This study used qualitative comparative analysis (QCA). Compared to traditional methods, QCA offers a series of advantages that made it appropriate for this study. Contrary to traditional multiple regression analysis, QCA relies on asymmetrical relationships overcoming the limitations that appear due to the linearity and complementary associations between variables [84]. The goal of traditional methods has been to analyze the effect of a single variable on a particular outcome. In this regard, QCA allows one to discover the combination of the antecedent conditions (independent variables) that lead to a given outcome (in this study, the internal development of technological eco-innovations). QCA entails equifinality, since different associations between variables can result in the same outcome [85]. Each one of the possible associations or combinations of variables is known as a recipe. QCA considers both the presence and the absence of antecedent conditions [86]. Moreover, it is an appropriate method for the analysis of the data of this study since it offers valid responses when using small-to-intermediate research designs [87].

In this study, two specific QCA methods have been employed: crisp-set qualitative comparative analysis (csQCA) and fuzzy-set qualitative comparative analysis (fsQCA). csQCA is used for binary variables (i.e., the company develops/does not develop internal product eco-innovation). csQCA calibration uses categorical conditions based on a dichotomy, assigning full membership (value of 1) and full non-membership (value of 0) to each condition. On the other hand, fsQCA is appropriate for variables with continuous values (i.e., the number of employees of a company). fsQCA categorizes the variables into meaningful groups of cases [88]. The cut-off values range from full membership (0.95) to full non-membership (0.05) with the 0.5 case representing the maximum ambiguity. Fuzzy logic calibration combines qualitative and quantitative methods and requires theoretical and substantive knowledge of the context [87,89].

After calibrating the variables, the analysis of the necessity is done. The goal is to identify if all, or nearly all, instances of the outcome have the same condition for some of the considered variables. A condition is necessary if its consistency is particularly high (>0.95) and its coverage is not too low (>0.5). The creation of the truth table is the next step. The truth table sorts the cases according to the combinations of the causal conditions they exhibit (2^k^ rows). It considers all logically possible combinations of conditions, even those without empirical instances, and assesses the consistency of the cases in each row with respect to the outcome. Each empirical case (i.e., company) corresponds to a configuration (a row of the truth table) depending on the antecedent conditions that it meets [87,90]. The next step is the reduction of the cases (rows) using algorithms. A version of the Quine-McCluskey algorithm is the most commonly used algorithm to perform the logical reduction of the statements [91]. By using Boolean algebra, QCA identifies the minimal set of causal conditions that are sufficient to produce the outcome. The goodness-of-fit of the row reduction depends on two criteria: coverage and consistence. Similarly, to the traditional R^2^ value, the coverage refers to the number of cases for which a configuration is valid. The consistency refers to the percentage of causal configurations with similar compositions that result in the same outcome value [84,92].

The starting point of the study is the consideration of the different factors stimulating product and process eco-innovations in companies [93]. For that reason, different models are proposed to evaluate those differences. First, a general model including all companies of the sample that developed internal/acquired technological eco-innovations was performed. After that, specific models, including the companies that developed internal/acquired product eco-innovations and internal/acquired process eco-innovations, were developed. Table 2 shows the description of the variables that were considered and the transformation values of the outcome and the antecedent conditions into fuzzy and crisp set terms.

## 4. Empirical Results and Discussion

The individual effect of each antecedent condition on the development of internal technological eco-innovation is shown in Table 3. The same data are shown in Table 4 for the internal development of product eco-innovations, and in Table 5 for the internal development of process eco-innovations. The antecedent conditions alone are insufficient for the outcome. In the case of the absence of the Group variable for product eco-innovation, a high value appears. This is the result of the small number of companies in the sample belonging to a company group. Moreover, as the value is lower than 0.95, the condition is not considered sufficient.

Table 6 shows the results of the model predicting the development of technological eco-innovation in agri-food companies. By using the notation introduced by Ragin and Fiss [94], black circles indicate the presence of the condition (●), white circles indicate the absence of the condition (○), and the absence of a circle indicates that the condition is not binding in that configuration. Up to four different recipes (configurations) result in the internal development of technological eco-innovations in companies. All of the paths (configurations 1 to 4, Table 6) require cooperation with internal and external partners, while the influence of R&D is not binding for most of the configurations. Previously, some studies have suggested that R&D is less important to eco-innovation when compared with collaboration strategies in the industrial sector [61]. The limited effect of R&D spending on technological eco-innovation was also reported by Cuerva, Triguero-Cano and Córcoles [1] who suggested that even though R&D promotes mainstream innovation, the case was not the same for eco-innovation.

Up to 27.8% of companies developing internal technological eco-innovations are small companies that neither belong to a company group nor adopt eco-organizational capabilities. They rely only on cooperation (configuration 1, Table 6). Effectively, cooperation enables the acquisition of complex and new knowledge required for eco-innovation. Moreover, cooperative food firms with high profit ratios also have a high probability of adopting internal eco-innovations (up to 25.6% of companies in configuration 2, Table 5). Cooperation improves firm efficiency and profits [76], and it is also considered an essential part of the open innovation concept [16]. Cooperation with partners has recently been identified as a driver for the development of eco-innovations in the manufacturing sector in general [95,96] and for the introduction of radical eco-innovations, specifically in the food and beverage sector [16]. Other conditions, such as firm size or technological capabilities, are only important in configurations that include companies that belong to a company group and have high capital ratio and/or profitability (configurations 3 and 4, Table 6), but the percentage of food firms is lower (around 2.7 and 3.2 percent, respectively). Although some studies show that firm size has a positive influence on eco-innovation, the empirical evidence is not all conclusive. Firm size has been identified as crucial, but also as an indeterminate factor in explaining eco-innovations in the manufacturing industry [3,93]. This result does not mean that size or R&D do not have an influence on the adoption of internal eco-innovation by food companies, but it shows that cooperation has a more essential role than financial and technological capabilities related to size.

The model with the configurations resulting in the development of internal product eco-innovation is shown in Table 7. The coverage value of the model is high (0.56), meaning that the model is valid for a large number of agri-food companies. Up to seven different configurations lead to the internal development of the less frequent type of technological eco-innovation in the food industry. Configurations 1 and 4 have high raw coverages, and thus deserve further attention since they explain the conditions of more companies (14.6 and 14.8%, respectively). Configuration 1 includes small companies that do not belong to a company group and have low profitability. However, they succeed in developing internal product eco-innovations through their eco-organizational capabilities and knowledge cooperation. These firm capabilities with sufficient capital availability foster the adoption of product eco-innovations. As pointed out by Dora et al. [97], eco-organizational capabilities related to quality assurance methods, such as food safety, Hazard Analysis and Critical Control Points (HACCP), British Retail Consortium (BRC), International Organization for Standardization (ISO), or microbiological issues, are directly linked to product innovations in the food sector. Cooperation is also crucial for the adoption of product eco-innovations by SMEs due to the low technological opportunities by SMEs compared to large companies [14]. Higher levels of financial performance and R&D capabilities have been also previously proposed as drivers of the internal development of eco-product innovations [73,74,98]. Hence, the introduction of novel eco-products in the food industry, such as functional foods, plant-based meats, or foodstuffs using genetic engineering are normally carried out by large food companies with corporate profitability and R&D departments. In configuration 4, high profitability and R&D expenditure replace a high capital ratio in configuration 1, explaining how these factors predict the adoption of eco-product innovation by about 14.7% of the sample. The adoption of eco-process innovations interacts with other factors to explain the development of eco-product innovations: large firms with high profitability that cooperate and adopt eco-organizational innovations (configuration 2); firms with high profitability that cooperate and adopt eco-organizational innovations (configuration 3); and firms with high profitability and capital ratios that cooperate (configuration 5). The two former configurations show how the complementarity of eco-organizational innovations and eco-process innovations explain the adoption of eco-product innovations, while the latter shows the role of capital in adopting eco-process innovations that enhances eco-product innovations. The first result is in line with the existence of complementarities across the different types of eco-innovation activities shown in the related literature [2,4], as well as the positive influence of proactive environmental management and incremental organizational eco-innovations in the adoption of eco-innovations by Spanish food firms [99]. The second one indicates the capital requirements needed by the food industry to do eco-process innovations, often related to the acquisition of new machinery and equipment [16].

To summarize, all configurations need cooperation to develop internal product eco-innovations. The importance of external sources of knowledge and information in the development of eco-innovation activities has been previously illustrated by Horbach [3]. However, other conditions are also important, since they appear in most of the configurations for internal product eco-innovations. This is the case for eco-organizational capabilities, high corporate profitability, and the development of process eco-innovations.

Table 8 shows the model predicting the development of internal process eco-innovations in agri-food companies. The solution consistency of the models ranges from 0.84 to 1.00, which are higher than the minimum value (0.8) recommended by Ragin [88]. Similar to the results for the companies developing internal product eco-innovations, the configuration that includes most of the companies (28.5% of the sample) relies only on cooperation with partners (configuration 1). Previously, cooperation had been identified as the main driver of continuous process innovation in a review covering the fertilizer and agricultural sector and other related studies [100].

Unlike other types of eco-innovation, the absence of eco-organizational innovations and product eco-innovation in food companies is not a constraint to developing internal process eco-innovation in the resource and capability combinations that include most of the companies (configurations 1 and 2, Table 8). The development of process eco-innovations is considered less demanding of complementary eco-innovations related to non-technological and technological capabilities than product eco-innovations.

Since innovation in the agri-food sector is mostly incremental [101] and relies mainly on external knowledge through cooperation [21], the effect of company R&D spending on internal technological eco-innovations was expected to be limited, compared to the results obtained for Spanish manufacturing firms in general [102]. Previously, some studies have shown the limitations of R&D for promoting eco-innovation in the sector [1]. Hence, the same results are obtained for technological eco-innovation in general (Table 6). However, the joint use of R&D and cooperation for food firms (configuration 2) is also identified by about 21.0% of process eco-innovators. This result shows that about one-fifth of companies adopting process eco-innovations also invest in R&D, but in a complementary mode to cooperate with external actors. Similarly, cooperation interacts with the rest of the financial, technological, and eco-organizational capabilities to predict the development of internal process eco-innovations (configurations 3, 4, and 5).

To sum up, most food firms adopting internal product and process eco-innovation depend on cooperation. However, our empirical models show different configurations to predict the development of each type of eco-innovation. Results are different when the sample is divided into internal product and process eco-innovations. Once the sample is divided, high R&D spending can be considered a beneficial condition to developing both product eco-innovations (configurations 4 and 6, Table 7) and process eco-innovations (configurations 2 and 4, Table 8) under specific circumstances. Financial capabilities related to size, profit, and capital also interact in a different way to predict each eco-innovative path. However, the most insightful result is regarding the complementarities between eco-organizational capabilities in each type of eco-innovation. Specifically, the adoption of eco-process innovations (technological capabilities) and eco-organizational capabilities are relevant for product eco-innovation (Table 7). Although this type of eco-innovation also depends on market pull factors [4], these findings make a lot of sense. On one hand, the implementation of process eco-innovations in the upstream stages must lead to the development of downstream product eco-innovations. On the other hand, eco-organizational capabilities enhance the internal development of product eco-innovations, probably because environmental management strategies in the food industry (such as food safety and quality systems or labelling) also contributes to the success of product eco-innovations. In particular, some certifications and labels (i.e., EMS) provide valuable information to the consumer affecting their confidence about new products in a traditional sector, such as the food and beverage industry [103]. However, the opposite does not apply for process eco-innovations. Product eco-innovations are not a crucial factor to the development of internal process eco-innovation by agri-food companies, where cooperation and R&D are more relevant (Table 8).

## 5. Conclusions

Although the number of studies about eco-innovation has significantly increased in the last decade [2], some aspects about companies’ eco-innovation drivers and conditions remain unclear. In general, studies have focused on the whole industrial sector [61,93,96], with few paying attention to specific sectors, such as the agri-food industry [16]. Regarding specific eco-innovations, the differences between product and process eco-innovations are persistent in the current studies. In this scenario, this study went beyond that differentiation and analyzed the conditions that promote internal technological eco-innovations, distinguishing between products and processes within a company. To achieve this, a new method (QCA) with proven guarantees in the business and management area has been used [92].

The study showed that the proposed conditions are useful for explaining the development of internal technological eco-innovations in the agri-food industry. All models and configurations concur with the idea that cooperation with external partners is the key to success in the development of internal technological eco-innovations in general, and internal product and process eco-innovations, more specifically. It must be considered that the agri-food sector is mainly composed of SMEs, and empirical evidence has found that these small companies can obtain the best results in developing technological eco-innovations through the recipe of cooperation. This conclusion encourages the design of policies and incentives to promote cooperation between companies operating in the agri-food industry so as to enhance innovative patterns and make them more environmentally friendly, which also allows for the increase of competitive advantages and the efficient use of capabilities and limited resources by small firms operating in a traditional low-tech sector.

Although similarities in the conditions of developing product and process eco-innovations have appeared, some differences also exist. Companies that develop eco-organizational capabilities and process eco-innovations tend also to develop product eco-innovations. However, these conditions do not apply to the development of process eco-innovations. This may be the result of the stage of production in which each type of eco-innovation is made. Upstream eco-innovation (process) encourages the development of subsequent eco-innovations (product), but the opposite does not apply. This analysis of the effects of the individual types of eco-innovation provides important information regarding the design and planning of eco-innovation strategies by SMEs. According to our findings, firms should cooperate with external partners to gather the exploitation of inbound information flow that is valuable to eco-innovation. In addition, firms enabling access to necessary knowledge through these cooperative relationships can improve their eco-processes and eco-products. At that point, the adoption of process eco-innovations can develop skills and capabilities that can be used to improve and introduce eco-products. According to our results, food companies that are more committed to eco-process and eco-organizational changes are also more likely to introduce product eco-innovations, taking advantage of the complementarity and synergies derived from open innovation schemes in an industry based on natural resources, such as the agri-food industry. In this regard, our findings are in line with the previous literature showing that the integration of potential solutions for the production stage (eco-process), the consumption stage (eco-products), and the production-supply-disposal chain (eco-organizational) can help in the transition towards a circular system and more sustainable innovative practices in the food industry [104].

Limitations in this study also appeared, due to the low number of companies that were analyzed. Due to the outcome’s specificity, the number of companies that was considered in the analysis of each outcome within the original sample was inevitably small. However, this limitation is considered to have been solved by using QCA. Although the coverage and the consistency of the models is adequate, the question regarding whether the variables that were considered in this study were the best proxies for capturing the development of the technological eco-innovations in the sector remains unanswered.

## Figures and Tables

**Table 1:** Highly cited papers on eco-innovation from the Web of Science, 2017–2019.

Authors	Comments
Mavi et al. (2019) [28]	Empirical analysis on the joint effect of eco-efficiency and eco-innovation on economic growth
Zhang et al. (2019) [29]	Empirical analysis on the effect of eco-innovation on performance
Kusi-Sarpong et al. (2019) [30]	Empirical analysis on sustainable supply chains in manufacturing companies
Stucki (2019) [31]	Empirical analysis on the effect of eco-innovation (green energy) on performance
Saidani et al. (2019) [26]	Literature review on eco-innovation and circular economy indicators
Kiefer et al. (2019) [32]	Resources and capabilities as drivers of different eco-innovations
Díaz-Lopez et al. (2019) [33]	Empirical analysis on the implementation of resource efficiency measures
Kirchherr et al. (2018) [34]	Empirical analysis on circular economy barriers
Li et al. (2018) [35]	Empirical analysis on the role of eco-innovation as a mediator on corporate carbon disclosure
Prieto-Sandoval et al. (2018) [27]	Literature review on eco-innovation as a precursor of circular economy
Ben Arfi et al. (2018) [36]	Empirical analysis on the role of external knowledge as driver of eco-innovation
Yuan and Xiang (2018) [37]	Empirical analysis on the role of regulation as a driver of eco-innovation
De Jesus and Mendonca (2018) [38]	Literature review on the drivers of eco-innovation and circular economy
Cai and Li (2018) [39]	Empirical analysis on the drivers of eco-innovation and its impact on performance
Tang et al. (2018) [40]	Analysis of the moderating role of management on the effect of eco-innovation on performance
Watson et al. (2018) [41]	Literature review on engaging stakeholders in environmental innovation
Choi (2018) [42]	Analysis of the interactions between technology-push and demand-pull factors and the role of industry life cycles and domestic market status in the electric vehicle sector
Feng and Chen (2018) [43]	Analysis of the role of environmental regulation in the impact of green innovation on industrial green development
Huang and Li (2017) [44]	This paper identifies the factors influencing green innovation and the relationship between green innovation and performance
Gupta and Barua (2017) [45]	The paper presents a framework for supplier selection by large companies considering green innovation
Costantini et al. (2017) [46]	Empirical analysis of the role played by selected characteristics of the policy mix in inducing innovation in energy efficiency technologies
Beltran-Esteve and Picazo-Tadeo (2017) [47]	The paper assesses environmental performance in the European Union using Luenberger productivity indicators, directional distance functions and Data Envelopment Analysis techniques
Jansson et al. (2017) [48]	Analysis of the relationship between market orientation and entrepreneurial orientation in relation to sustainability practices in SMEs
Zhang et al. (2017) [49]	Estimation of the effect of environmental innovation on carbon emissions in China
Notarnicola et al. (2017) [50]	Analysis of the environmental impact of food consumption using a lifecycle assessment approach

Highly Cites Papers are those papers cited on top 1% of their field in their year of publication. Source: Own elaboration.

**Table 2:** Variable definitions and calibration values.

Condition	Description	Membership Threshold Values		
		Full Non-Membership (0.05)	Crossover Point (0.5)	Full Membership (0.95)
Internal technological Eco-Innovation	The company develops an internal product or process eco-innovation	0		1
Internal Product Eco-Innovation	The company develops an internal product eco-innovation	0		1
Internal Process Eco-Innovation	The company develops an internal process eco-innovation	0		1
Eco-Organizational Capabilities	The company develops a non-technological eco-innovation (marketing, organizational)	0		1
Group	The company is part of a company group	0		1
Cooperation	Number of internal or external partners the company cooperates with in the development of eco-innovations	0	0.95	2
R&D	R&D expenditures as a percentage of sales	0	1.95	15
Size	Number of employees	4.9	76.0	361.4
Capital	Company capital (thousands of euros)	3	1125	21219.5
Profitability	Company profit margin (%)	−5.77	3.47	15.28

**Table 3:** Companies that developed internal technological eco-innovations (any type). Analysis of the necessary conditions.

Conditions Tested *	Consistency	Coverage
Group	0.090909	1.000000
~Group	0.909091	0.533333
Eco-Organizational Capabilities	0.454545	0.606061
~ Eco-Organizational Capabilities	0.545455	0.521739
Cooperation	0.752500	0.839503
~Cooperation	0.247500	0.275278
R&D	0.457955	0.647286
~ R&D	0.542046	0.498224
Size	0.417045	0.523538
~Size	0.582955	0.583618
Capital	0.359964	0.494210
~Capital	0.641136	0.599575
Profitability	0.491591	0.584121
~Profitability	0.508409	0.533000

* The symbol (~) represents the negation of the characteristic.

**Table 4:** Companies developing internal product eco-innovations. Analysis of the necessary conditions.

Conditions Tested *	Consistency	Coverage
Group	0.058824	1.000000
~Group	0.921176	0.761905
Eco-Organizational Capabilities	0.588235	0.769231
~ Eco-Organizational Capabilities	0.411765	0.666667
Cooperation	0.732941	0.980330
~Cooperation	0.267059	0.488698
R&D	0.419412	0.839811
~ R&D	0.580588	0.730570
Size	0.357647	0.784516
~Size	0.642353	0.766316
Capital	0.454118	0.845564
~Capital	0.545882	0.721057
Profitability	0.442941	0.726133
~Profitability	0.442941	0.726133
Process eco-innovation	0.529412	0.900000
~ Process eco-innovation	0.470588	0.666667

* The symbol (~) represents the negation of the characteristic.

**Table 5:** Companies developing internal process innovations. Analysis of the necessary conditions.

Conditions Tested *	Consistency	Coverage
Group	0.093750	1.000000
~Group	0.906250	0.460317
Eco-Organizational Capabilities	0.406250	0.500000
~ Eco-Organizational Capabilities	0.593750	0.475000
Cooperation	0.755313	0.747603
~Cooperation	0.244688	0.232551
R&D	0.500938	0.603085
~ R&D	0.499062	0.405124
Size	0.409375	0.438714
~Size	0.590625	0.522966
Capital	0.292500	0.356165
~Capital	0.707500	0.569990
Profitability	0.470000	0.514716
~Profitability	0.530000	0.461120
Product eco-innovation	0.156250	0.555556
~ Product eco-innovation	0.843750	0.473684

* The symbol (~) represents the negation of the characteristic.

**Table 6:** Model predicting the development of internal technological eco-innovations (product or process) in agri-food companies.

Configurationo.	Group	Eco-Organ. Capab	Cooperation	R&D	Size	Capital	Profitability	Coverage		Consistency
								Raw	Unique	
**1**	○	○	●		○	○		0.2780	0.1259	0.9154
2	○		●		○	○	●	0.2564	0.1043	0.8558
3	●		●	○	○	●	●	0.0266	0.0172	1.0000
4	●	●	●		●	○	●	0.0316	0.0223	1.0000
Solution coverage: 0.4311										
Solution consistency: 0.9068										

Eco-Organizational Capabilities (Eco-Organ. Capab.). Frequency threshold = 1, and consistency threshold = 0.8186.

**Table 7:** Model predicting the development of internal product eco-innovations in agri-food companies.

Configuration no.	Group	Eco-Organ Capab	Cooperation	R&D	Size	Capital	Profitability	Process Eco-Innovation	Coverage		Consistency
									Raw	Unique	
**1**	○	●	●		○	●	○		0.1464	0.0841	0.9614
2	○	●	●		●		○	●	0.0894	0.0506	0.9682
3	○	●	●		○	○	●	●	0.0865	0.0047	0.9671
4	○	●	●	●	○	○	●		0.1477	0.0580	0.9436
5	○	○	●		●	●	●	○	0.1018	0.1018	0.9454
6	○	○	●	●	○		○	●	0.1165	0.1165	1.0000
7	●	○	●	○	○	●	●	○	0.0312	0.0311	1.0000
Solution coverage: 0.5635											
Solution consistency: 0.9746											

Frequency threshold = 1, and consistency threshold = 0.8630.

**Table 8:** Model predicting the development of internal process eco-innovations in agri-food companies.

Configuration no.	Group	Eco-Organ Capab.	Cooperation	R&D	Size	Capital	Profitability	Product Eco-Innovation	Coverage		Consistency
									Raw	Unique	
**1**	○	○	●		○	○		○	0.2853	0.1322	0.8986
2	○	○	●	●	○	○	○		0.2097	0.0566	0.8483
3	●	●	●		●	○	●	○	0.0434	0.0306	1.0000
4	○	●	●	●	○	●	○	●	0.0200	0.0200	0.8530
5	●	●	●	○	○	●	●	○	0.0200	0.0072	1.0000
Solution coverage: 0.4126											
Solution consistency: 0.9097											

Frequency threshold = 1, and consistency threshold = 0.8138.
